# Recent advances in understanding mitochondrial genome diversity

**DOI:** 10.12688/f1000research.21490.1

**Published:** 2020-04-17

**Authors:** Rafael Zardoya

**Affiliations:** 1 Departamento de Biodiversidad y Biología Evolutiva, Museo Nacional de Ciencias Naturales (MNCN-CSIC), Madrid, Spain

**Keywords:** mitochondrial genomes, animal, plant, fungi, unicellular eukaryotes

## Abstract

Ever since its discovery, the double-stranded DNA contained in the mitochondria of eukaryotes has fascinated researchers because of its bacterial endosymbiotic origin, crucial role in encoding subunits of the respiratory complexes, compact nature, and specific inheritance mechanisms. In the last few years, high-throughput sequencing techniques have accelerated the sequencing of mitochondrial genomes (mitogenomes) and uncovered the great diversity of organizations, gene contents, and modes of replication and transcription found in living eukaryotes. Some early divergent lineages of unicellular eukaryotes retain certain synteny and gene content resembling those observed in the genomes of alphaproteobacteria (the inferred closest living group of mitochondria), whereas others adapted to anaerobic environments have drastically reduced or even lost the mitogenome. In the three main multicellular lineages of eukaryotes, mitogenomes have pursued diverse evolutionary trajectories in which different types of molecules (circular versus linear and single versus multipartite), gene structures (with or without self-splicing introns), gene contents, gene orders, genetic codes, and transfer RNA editing mechanisms have been selected. Whereas animals have evolved a rather compact mitochondrial genome between 11 and 50 Kb in length with a highly conserved gene content in bilaterians, plants exhibit large mitochondrial genomes of 66 Kb to 11.3 Mb with large intergenic repetitions prone to recombination, and fungal mitogenomes have intermediate sizes of 12 to 236 Kb.

## Introduction

Mitochondria are specialized organelles of eukaryotic cells in charge of essential roles, including the supply of energy (in the form of ATP) through aerobic respiration (oxidative phosphorylation, or OXPHOS), the biosynthesis of different types of lipids and iron–sulfur clusters, programmed cell death (apoptosis), calcium homeostasis, and the reaction to stressors, among many others ^1^. The most intriguing feature of mitochondria is arguably their endosymbiotic origin ^2^ (for a recent retrospective, see 3). All mitochondria descend from an ancestor related to extant alphaproteobacteria ^4^ which integrated into a host cell related to extant Asgard archaea ^5^. However, owing to potential biases affecting phylogenetic inference, the precise closest living sister groups of neither the symbiont nor the host are known ^6^. Though still under debate ^7^, the integration likely occurred late during eukaryogenesis ^8^. The endosymbiosis was a complex evolutionary process, which likely implied incremental steps, including the acquisition of the protein and RNA import machinery, the modification of the endosymbiont membranes, the massive gene transfer to the nucleus, the forming of the inner membrane invaginations, the coordination of biochemical pathways, and the integration of mitochondrial division into the cell cycle ^6^. As a result, mitochondria exhibit mosaic proteomes with about 1000 proteins of mixed evolutionary origins ^9^. Most proteins of alphaproteobacterial origin are involved in aerobic respiration and ribosomal function, whereas proteins of eukaryotic origin are generally in charge of nuclear–mitochondria signaling ^6^.

Despite the massive loss (due to functional redundancy) or transfer of genes to the nucleus during the endosymbiosis of mitochondria, these organelles still retain a reduced set of protein-coding genes that are replicated, transcribed, and translated independently from the nuclear genome. Remarkably, these mitochondrial protein-coding genes are translated using genetic codes different from the standard (nuclear) genetic code in many living eukaryotes ^10^. Several hypotheses have been postulated to explain the evolutionary persistence of a mitochondrial genome (hereafter mitogenome) in eukaryote cells despite the evolutionary pressure to centralize genetic information in the nucleus ^11^. Proteins of the respiratory complexes encoded by the mitogenome are highly hydrophobic and thus would be difficult to import and insert into the inner mitochondrial membrane if produced in the cytosol. In addition, the possibility of mis-targeting the proteins to the endoplasmic reticulum would be a crucial problem to solve ^11^. Theoretically, it is also conceivable that co-location of genes in the same compartment as their gene products could facilitate direct regulatory coupling and redox control ^12^.

Mitogenomes are double-stranded DNA molecules of variable size that generally are found as circular, linear, or branched forms ^13^. Each cell may contain more than 1000 mitogenomes (the so-called chondriome), showing length and site heteroplasmy (nucleotide [nt] variation) and allowing the accumulation of mutations without immediate deleterious effects ^14^. The mitogenome population of an individual is constantly varying and subjected both to genetic drift, as some mitogenomes may segregate more frequently than others by chance, and to selection, as those molecules that provide higher energy production or that replicate more often may be transmitted more efficiently ^15^. Genetic bottlenecks and recombination can also have profound effects on heteroplasmy. The relative influence of genetic drift and selection on mitogenomes depends on their inheritance mechanism, which varies among animals, fungi, and plants ^16^. Maternal inheritance of mitogenomes due to selective degradation of paternal ones is the prevalent mechanism in animals and plants ^17^, although remarkable exceptions of paternal leakage producing doubly uniparental inheritance (DUI) have been reported in, for example, mussels ^18^, the bladder campion, *Silene vulgaris* ^19^, and very exceptional cases in humans ^20^. In fungi, there are a variety of inheritance patterns during sexual reproduction ^21– 23^. In Ascomycota yeasts, biparental inheritance of mitochondria in the zygote is common, but later on, one of the parental mitogenomes is usually eliminated ^21^. In filamentous Ascomycota, mitochondria are generally inherited from the larger of the two cells involved in the mating process ^22, 23^. In Basidiomycota, both uniparental and biparental inheritance modes are reported ^21^.

The replication ^24^ and transcription ^25^ of mitogenomes have been best studied in vertebrates as the original experimental studies to identify the different proteins were performed in this group ^26, 27^. The control region of the vertebrate mitogenome has the origin of replication of one strand and the origins for transcription of both strands, whereas the origin of replication for the opposite strand is located within a cluster of transfer RNA (tRNA) genes between the *cox1* and *nad2* genes ^28^. The traditional strand displacement model for replication of mammal mitogenomes implies initiation from the origin of replication using a transcript as primer. After replication of the first strand exposes the origin of replication of the lagging strand, its replication is initiated ^24, 28^. Alternative models of strand-coupled replication involving the incorporation of RNA fragments in the newly synthesized lagging strand (implying the formation of Okazaki fragments) have been also proposed ^29^. The enzyme in charge of mitogenome replication in mammals is the nucleus-encoded DNA polymerase γ, which belongs to the family A DNA polymerases and contains a proofreading 3′–5′ exonuclease ^24, 28^. In plant and fungi mitogenomes, a recombination-dependent or rolling circle mechanism similar to bacteriophage T4 DNA replication or both have been proposed ^30^. Whether there are specific origins of replication and their putative location are not known. The enzymes in charge of mitogenome replication in *Arabidopsis* are DNA polymerase IA and IB, also belonging to the family A DNA polymerases ^30^. Whereas animal mitogenomes have been shown to evolve faster than their nuclear counterparts, the mitogenomes of plants and fungi evolved generally slower than the corresponding nuclear genomes ^22^. It has been postulated that the different replication models of plant and fungal versus vertebrate mitogenomes imply different repairing capacities that affect fidelity of the copies, which may explain main differences in evolutionary rates ^31^.

The enzyme in charge of transcription is a DNA-dependent RNA polymerase related to RNA polymerases in T3 and T7 bacteriophages ^25^. A long primary polycistronic transcript is synthesized and later cleaved by the RNase P and RNase Z at the 5′ and 3′ ends of the intervening tRNAs, respectively ^25^. This “tRNA punctuation model” cannot explain the processing of the primary transcript outside vertebrates, as tRNA genes in other eukaryote mitogenomes either are frequently grouped together in clusters or simply have been massively lost. Alternatively, in humans, Fas-activated serine/threonine kinases (FASTKs) have been shown to be involved in the processing of transcript precursors derived from adjacent genes that lack an intervening tRNA ^32^. These proteins have a conserved C-terminal RAP domain, which might have a putative endonuclease activity and could represent a more general (outside vertebrates) mitochondrial RNA processing mechanism ^25^. After cleavage, a poly-A tail is added to the 3′ end of the individual messenger RNAs (mRNAs) by a polyadenylic acid RNA polymerase. This polyadenylation is crucial as it completes the stop codons of several genes.

The tRNA maturation involves a chemical modification by several enzymes of the first position of the tRNA anticodon to facilitate non–Watson-Crick (wobble) base pairing ^25^. This is critical as the tRNA repertoire of mitogenomes is often reduced and the modification expands codon recognition during mitochondrial translation. In addition, editing of the aminoacyl acceptor stems is a widespread mechanism occurring during the post-transcriptional processing of mitochondrial tRNAs ^33^. Finally, it is important to note that, in some species, the existence of a reduced set of tRNA genes in the mitogenome is compensated by the import of nucleus-encoded tRNAs from the cytosol. These striking differences in tRNA processing and maturation result in deviations from the standard genetic code ^34^. Translation of mRNAs occurs in the mitochondrial ribosomes. Each mitoribosome is formed by a small subunit (SSU), which binds mRNAs and tRNAs, and a large subunit (LSU), which catalyzes the formation of peptide bonds. Several of the proteins (except in animals) and the SSU and LSU rRNAs that form both subunits are encoded by mitogenomes. In some species, the mitogenome also encodes the 5S rRNA. Other non-coding RNAs such as the H1 RNA (the RNA component of the RNAseP), the RMRP (involved in 5.8S RNA processing), the hTERC (the RNA component of telomerase), and various microRNAs are encoded by the nuclear genome and imported into the mitochondria ^35^. In mammals, RNA import is ATP-dependent and requires the presence of the membrane potential, a specific channel in the outer mitochondrial membrane, and the involvement of the polynucleotide phosphorylase (PNPASE), a 3′-to-5′ exoribonuclease and poly-A polymerase located in the mitochondrial intermembrane space ^35, 36^. Mitoribosome assembly is performed at the so-called mitochondrial RNA granules ^25^. In the mitochondria, the mitogenomes are not present as naked DNA but packaged with proteins (the most essential is the mitochondrial transcription factor A, or TFAM) in the so-called nucleoids, which are required for correct mitogenome replication, transcription, and translation ^37^.

**Figure 1.**
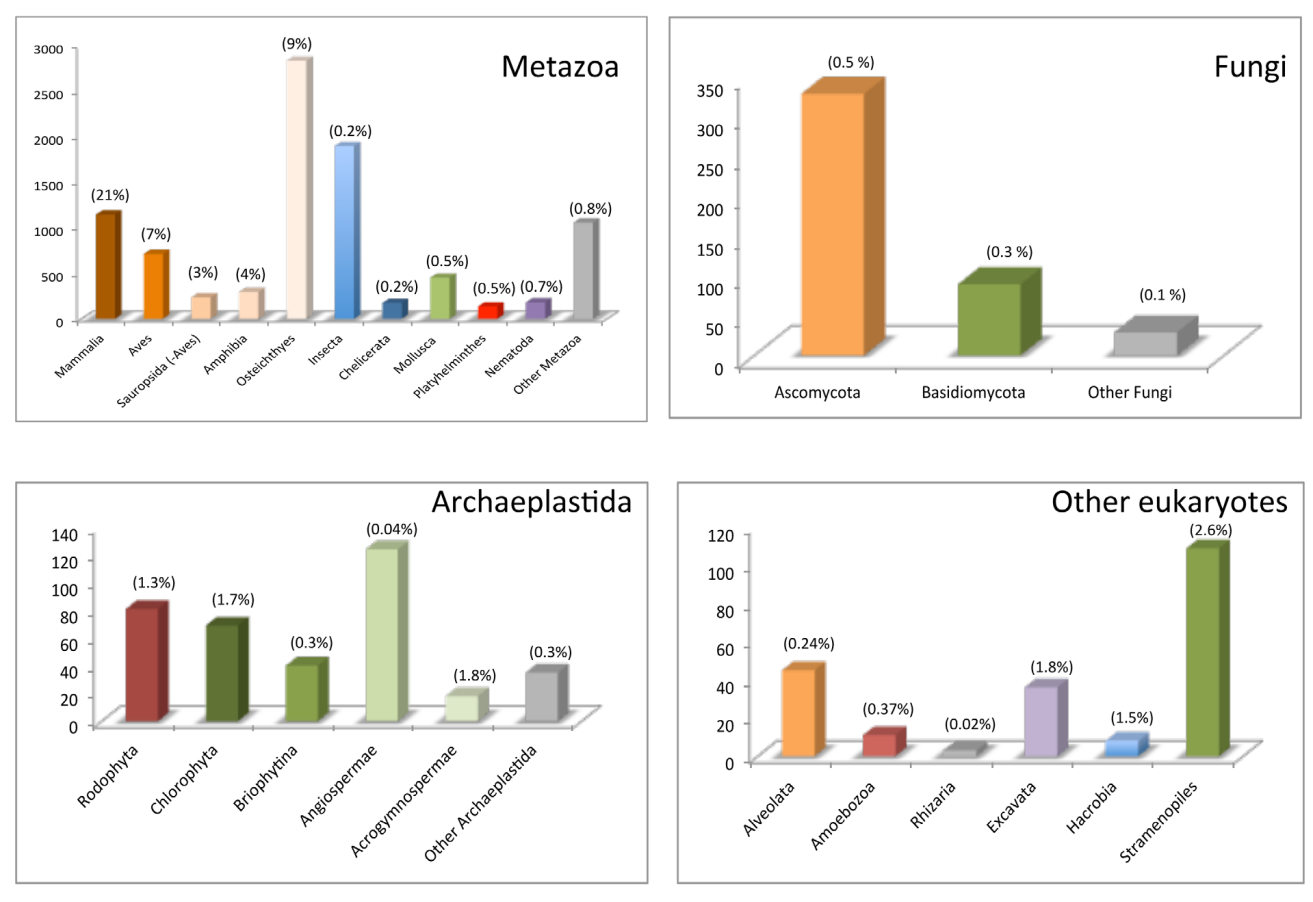
Currently sequenced complete mitogenomes. Complete mitogenome sequences are available at the organelle genomes database, which is part of the National Center for Biotechnology Information Reference Sequence project. Release 7/11/19 was downloaded and mitogenomes were separated by groups (Metazoa, Fungi, Archaeplastida, and other eukaryotes). The proportion (percentage) of sequenced mitogenomes to the estimated number of species per group ^53^ is shown above each bar.

In the last several years, the development of high-throughput sequencing techniques has enormously accelerated the sequencing of mitogenomes (there were over 10,000 reference sequences in the National Center for Biotechnology Information [NCBI] organelle genome database as of November 2019; Figure 1), widening our view of their diversity. Here, I will review the most recent data available on the structure and gene content of the mitogenomes of the different groups of living eukaryotes within an evolutionary context.

## 1. Animal mitochondrial genome structure

**Figure 2.**
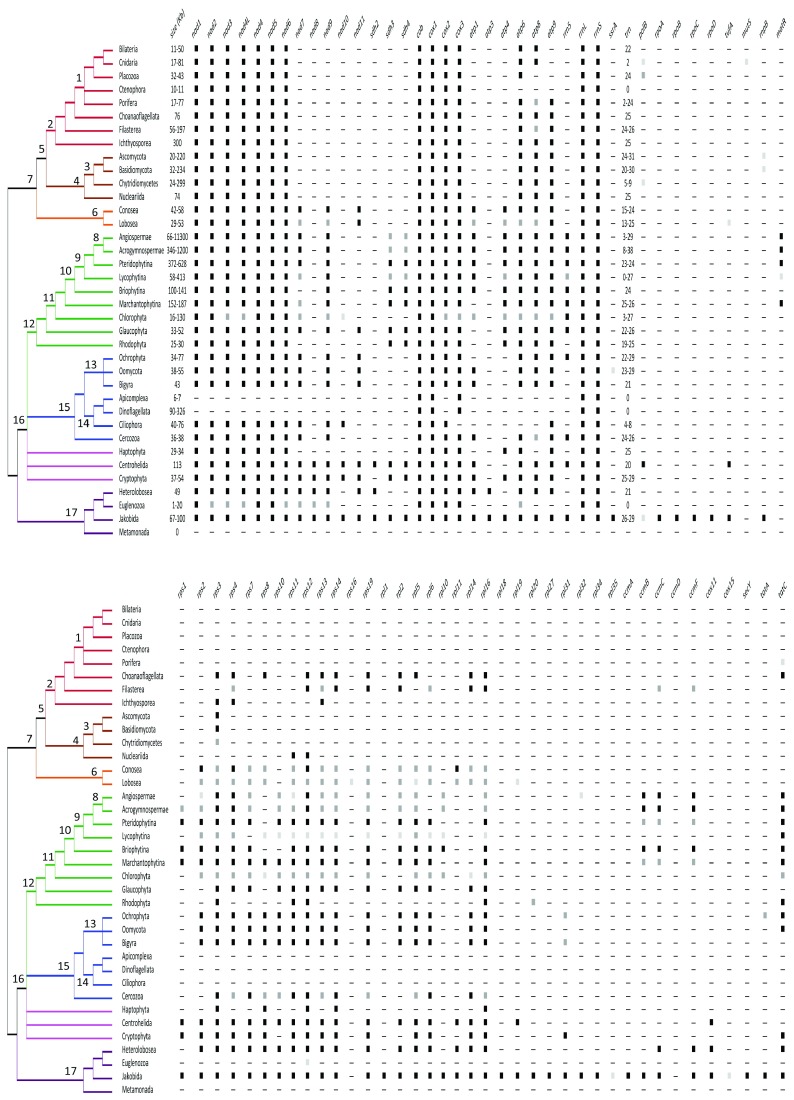
Gene content of mitogenomes. The mitogenome size (in kilobases) and the presence of the different mitochondrial genes are shown. The black, dark, and light gray squares indicate that the gene was reported in all or almost all, most, or few taxa of the group, respectively. For tRNA genes, the number is provided. A consensus phylogeny of the eukaryotes ^68^ is depicted. Numbers in the nodes refer to the following: 1. Metazoa; 2. Holozoa; 3. Fungi; 4. Holomycota; 5. Ophistokonta; 6. Amoeobozoa; 7. Amorphea; 8. Spermatophytes; 9. Tracheophytes; 10. Embryophytes; 11. Viridiplantae; 12. Archaeplastida; 13. Stramenolipes; 14. Alveolata; 15. SAR; 16. Diaphoreticktes; 17. Excavata ^68^.

The mitogenomes of bilaterian animals are extremely compact ( Figure 2). They are normally organized into a single circular molecule of about 16 Kb (ranging from 11 to 50 Kb) in length. Remarkably, however, in dicyemids (rhombozoans) and some rotifers, nematodes, thrips, and lice, the mitogenome is divided into multiple circular mini-chromosomes ^38^. Bilaterian animal mitogenomes normally contain 13 protein-coding genes ( *nad1–6*, *4L*; *cob*; *cox1–3*; and *atp6* and *8*), which encode different subunits of the enzyme complexes of the OXPHOS system; two rRNA genes; and 22 tRNA genes ( Figure 2). As an exception, the *atp8* gene is absent in most nematodes ^38^ and flatworms ^39^ and has been difficult to annotate in some bivalves ^40^. Furthermore, the mitogenome of the only species of arrow worm (phylum Chaetognatha) thus far sequenced lacks the *atp6* and *8* genes and all tRNA genes but *trnM* ^41^. All bilaterian mitochondrial genes lack introns, and the only reported exception is the *cox1* gene of one annelid ^42^. In general, genes abut with almost no intergenic regions, and the only exceptions are the so-called control regions that contain the origins of replication and transcription. The synteny is rather conserved within the different bilaterian phyla, although some (for example, Mollusca ^43^) are more prone to gene rearrangements than others (for example, Arthopoda ^44^). Within Chordata, the mitogenome organization of vertebrates is highly conserved whereas that of ascidians is hypervariable ^45^. Gene rearrangements between relatively closely related taxa usually involve *trn* genes (but not only) and are particularly frequent around the control regions, through the tandem duplication and random loss (TDRL) mechanism, which explains translocations ^46^ but not inversions.

The structures of the mitogenomes of the earliest lineages of metazoans (that is, Porifera, Ctenophora, Placozoa, and Cnidaria) differ variously from the above described for bilaterian animals ( Figure 2). Whereas the mitogenome of Ctenophora is 10 to 11 Kb in length, those of most of Cnidaria and Porifera are 16 to 20 Kb (although some sea anemones and calcareous sponges can reach up to 77 to 81 Kb) and that of Placozoa is 32 to 43 Kb ^47^. One notable difference is the presence of linear mitogenomes in calcareous sponges and several cnidarians ^48, 49^. In some cnidarians, the linear mitogenome is organized into a single chromosome with terminal inverted repeats ^47^, whereas in other cnidarians ^49, 50^ and calcareous sponges ^48^, there are multipartite mitogenomes. A second striking feature of the mitogenomes of non-bilaterian animals is the different gene content and the presence of introns in some genes. Additional mitochondrial protein-coding genes reported in non-bilaterian animal taxa are the following: *atp9* in most sponge mitogenomes; *polB* encoding a family B DNA polymerase in the placozoan and some jellyfish mitogenomes; *mutS* encoding a mismatch repair protein in the mitogenomes of some corals; and *tatC* encoding the twin-arginine translocase subunit C in the mitogenome of the sponge family Oscarellidae ^47^ ( Figure 2). On the other hand, ctenophores, placozoans, and calcareous sponges lack the *atp8* gene. The number of *trn* genes also differs between non-bilaterian and bilaterian animal mitogenomes. Those of demosponges and placozoans have in addition the *trnI(CAU*) and *trnR(UCU)* genes, whereas cnidarian mitogenomes (and independently one group of sponges ^51^) have reduced their set to only *trnM* and *trnW* ^47^ and those of ctenophores have lost all of their *trn* genes ^52^. The presence of self-splicing introns of groups I and II (typical of bacteria and viruses) has been reported in *cox1* and *nad5* genes of different species of corals, sponges, and placozoans ^47^. Group I and II introns contain internal *heg* (encoding homing endonucleases) and *matR* (encoding reverse transcriptases) genes, respectively ^54, 55^. It is postulated that the introns and many of the extra protein-coding genes (except *atp9* and *tatC*) were likely acquired through horizontal transfer ^47^.

Metazoa and their unicellular closest sister groups form the Holozoa. Within non-metazoan Holozoa, the mitogenomes of the choanoflagellate *Monosiga brevicollis*, the filasterean *Capsaspora owczarzaki* and *Ministeria vibrans*, and the ichthyosporean *Amoebidium parasiticum* have been sequenced ^56^. These mitogenomes are relatively large: 76, 197, 56, and 300 Kb, respectively. The variation in length is due to differences in repeats in non-coding regions. All are linear (with multiple chromosomes in *A. parasiticum*) except that of *M. brevicollis*, which is circular. All have the core set of genes found in bilaterian animals (except the *atp8* gene in *C. owczarzaki*) and the *atp9* gene found in the mitogenomes of sponges ( Figure 2). In addition, the mitogenomes of non-metazoan Holozoa have several *rps* and *rpl* genes encoding proteins of the small and large ribosomal subunits ( Figure 2). The *ccmC* and *ccmF* genes encoding proteins assisting in cytochrome *c* biogenesis are exclusive to *C. owczarzaki* ^56^, whereas the *tatC* gene is found only in *M. brevicollis*. The mitogenome of *A. parasiticum* is particularly rich in introns; there are 21 from group I and two from group II ^56^.

## 2. Fungal mitochondrial genome structure

Fungal mitochondrial DNAs are usually considered circular but may well exist as linear molecules *in vivo* (with inverted repeats at the ends ^57– 59^). Linearization has occurred independently several times in fungi (and other eukaryotes) and, according to one hypothesis, may have been triggered by the integration of linear mitochondrial plasmids often carrying the *dpoB* gene, which encodes a DNA polymerase B ^58, 60^. Fungal mitogenomes largely vary between 12 and 236 Kb ^57– 59, 61^ in length and are highly dynamic: important differences in sizes have been reported even between strains and individuals within the same species ^62– 64^. The variations in genome length and genetic diversity are attributed to differences in the number of introns, intergenic regions, and tandem repeats ^22, 65– 67^. Another remarkable feature of fungal mitogenomes is that they use several genetic codes. The typical mitochondrial genome of dikarya fungi (Ascomycota and Basidiomycota) contains 14 protein-coding genes ( *nad1*– *6*, *4L*; *cob*; *cox1*– *3*; and *atp6*, *8*, and *9*), the *rrnL* and *rrnS* genes, and a variable number (20 to 31) of *trn* genes ^22, 69^ ( Figure 2). The presence of the *rps3* (originally named *var1*) gene ^70, 71^ is widespread in dikarya fungal mitogenomes but is not present in all. A remarkable variation from the consensus is the lack of the *nad* genes in some yeasts (family Saccharomycetaceae) ^72^. A scattered distribution of the *rnpB* gene that encodes for the RNA subunit of the mitochondrial RNAse P is found in fungal mitogenomes ^69^. Genes are generally encoded in one strand in Ascomycota and in both in Basidiomycota. Introns are mostly of group I and normally contain *heg* genes ^22^. For instance, up to 43 group I introns have been reported in *Agaricus bisporus* ^73^. Gene rearrangements are frequent among dikarya mitogenomes and are most likely the result of intrachromosomal recombination events ^69^. The mitogenomes of early divergent lineages of fungi (for example, Mucoromycetes and Chytridiomycetes) have been less well studied ^74, 75^ ( Figure 1). The ones reported have the genes encoding the 14 OXPHOS complex subunits and the two rRNAs ^75^; some Mucoromycetes have, in addition, the *rnpB* and *rps3* genes ^74^; the *polB* gene as well as *heg* genes within group I introns have been found in the chytridiomycete *Synchytrium endobioticum* ^58^. An interesting feature of several of these mitogenomes is the drastic reduction in the number of *trn* genes to only five to nine in several chytridiomycete species such as *Hyaloraphidium curvatum* ^57^, *Monoblepharella* sp. ^76^, or *S. endobioticum* ^58^. Moreover, several of the mitogenomes of early divergent lineages of fungi have the *cox1* and *rrnS* genes divided into two fragments, separated by one or several genes, either at the same strand or at opposite strands ^75^.

The circular mitogenome of *Nuclearia simplex* of 74 Kb in length represents the Nucleariida, which are the unicellular sister group of fungi within Holomycota ^77^. This mitogenome has the same set of OXPHOS complex subunit genes typically found in fungal mitogenomes. In addition, it has the *rps11* and *12* genes ( Figure 2). The *rrnL* and *rrnS* genes and a total of 25 *trn* genes are present ^77^. Remarkably, this mitogenome contains a high number of introns (21 of group I and one of group II) and two to 10 unassigned open reading frames (ORFs).

## 3. Plant mitochondrial genome structure

In green plants (Viridiplantae), research effort has been preferentially concentrated in plastids; thus, the majority of the diversity of mitogenomes awaits further exploration ( Figure 1). Mitogenomes have been sequenced in angiosperms (for example, 78), acrogymnosperms ^79^, ferns ^80^, lycophytes ^81– 83^, mosses ^84– 86^, liverworts ^87^, and green algae ^88^ ( Figure 1).

The main feature that characterizes the mitogenomes of flowering plants (angiosperms) is their relatively large length, which varies a 100-fold range from 66 Kb of that of the parasitic plant *Viscum scurruloideum* ^89^ to 11.3 Mb of that of the eudicotyledon *Silene conica* ^90^. Within an angiosperm mitochondria, there could be several subgenomic isoforms with alternative arrangements resulting from homologous recombination that could have linear, branched, and circular structures ^91^. The angiosperm mitogenomes generally encode in both strands for a core set of 24 protein-coding genes: *nad1–7*, *9*, *4L*; *cob*; *cox1–3*; *atp1*, *4*, *6*, *8*, and *9*; the *ccmB*, *C*, *Fc*, and *Fn* genes encoding proteins assisting in cytochrome *c* biogenesis; the *tatC* (also referred to as *mttB*); and *matR* ^89^ ( Figure 2). In addition, angiosperm mitogenomes may have different combinations of *rps* and *rpl* genes as well as *sdh3* and *4* genes encoding succinate dehydrogenase subunits of OXPHOS complex II ^89^ ( Figure 2). Notably, the parasitic plant *V. scurruloideum* lacks all *nad* genes ^89^. Most angiosperm mitogenomes have homing group II introns in several protein-coding genes ^92^, whereas the presence of group I introns has been reported only in the *cox1* gene and has been inferred to result from an original horizontal gene transfer from fungi, and subsequent transfers occurred from one angiosperm to another ^93^. Angiosperm mitogenomes have the *rrn5* gene, which encodes the rRNA 5S of the large subunit in addition to the *rrnS* and *rrnL* genes. No angiosperm mitogenome has a complete set of *trn* genes and thus translation relies on nucleus-encoded tRNAs that are imported from the cytosol. In particular, *trnA*, *R*, *L*, *T*, and *V* genes are generally missing in angiosperm mitogenomes ^94^. The number of *trn* genes varies from only three to eight in *Viscum* ^89^ and *Silene* ^90^ to 17 to 29 genes in many species ^94^. Some of these *trn* genes have group I introns ^95^. Notably, a small fraction of the mitochondrial *trn* genes are derived from the chloroplast genome ^94^.

Variation in size of angiosperm mitogenomes is attributed mostly to differences in the number and length of non-coding regions ^96^ and to intracellular transfers from the nucleus and the chloroplast genomes ^90^. Differences in intron length ^97^ and horizontal gene transfer events ^98^ also account for size disparity. Intergenic regions in angiosperm mitogenomes are formed mostly by repeats of variable length well above 1 Kb. The dynamic nature of angiosperm mitogenomes relies mostly on large non-tandem repeats, which are responsible for rearrangements and changes in size through homologous recombination ^99^. The high recombination activity implies that gene order varies extensively even at the intra-specific level.

The core set of 24 protein-coding genes defined for angiosperms plus the three *rrn* genes are generally conserved in acrogymnosperms, ferns, spikemosses, mosses, liverworts, and green algae mitogenomes ( Figure 2). However, the number of *rps* and *rpl* genes varies substantially in the mitogenomes of these lineages. The number of introns within genes varies between 20 and 40 and most are of group II. Within acrogymnosperms, mitogenome research has been focused mostly on the early divergent lineages corresponding to genera *Cycas*, *Ginkgo*, and *Welwitschia* ^79^. As in angiosperms, the mitogenomes of the three above-mentioned acrogymnosperms are relatively large: 415, 346, and 979 Kb, respectively. The *Welwitschia* mitogenome has a reduced set of *trn* genes ^79^. The mitogenome of *Pinus taeda* is available at GenBank (MF991879; unpublished) and has the same protein-coding gene content found in *Cycas* and *Ginkgo* and many angiosperms, as well as 38 *trn* genes, which is higher than usual.

Thus far, the mitogenomes of *Ophioglossum californicum* and *Psilotum nudum* represent the ferns (Pteridophytina ^80^). They are 372 Kb (one circular chromosome) and 628 Kb (two circular chromosomes) in length, respectively ( Figure 2). The difference in length is due to repetitive intergenic sequences as the gene content of both species is virtually the same as that found in *Cycas* and *Ginkgo* and many angiosperms ^80^. The *nad* genes are particularly intron-rich and thus rather long. For instance, the *Psilotum nad1* gene is 21 Kb in length. The mitogenomes of spikemosses and quillworts (Lycophytina) have been sequenced in the three families within the group (that is, Selaginellaceae ^81^, Isoetaceae ^82^, and Lycopodiaceae ^83^). These genomes are 413, 58, and 183 Kb in length, respectively ( Figure 2). From the core set of 24 genes, all three representatives lack the *ccm* genes ^81– 83^ ( Figure 2). Also, *atp4* is missing in Selaginellaceae, *nad7* in Lycopodiaceae, and *matR* in Isoetaceae and Selaginellaceae. The set of accessory genes found in angiosperm mitogenomes is found almost complete in the Lycopodiaceae mitogenome ^83^ but heavily reduced in the Isoetaceae ^82^ and Selaginellaceae ^81^ mitogenomes ( Figure 2). The Selaginellaceae mitogenome lacks the *rrn5* gene and has no *trn* genes ^80^. The moss (Briophytina) mitogenomes are 100 to 141 Kb in length ^84, 85^. They lack the *matR* gene ( Figure 2). Synteny is largely conserved among moss mitogenomes ^85^. The mitogenomes of representatives of the three main liverwort lineages—Haplomitriopsida ^100^, Jungermanniopsida ^101, 102^, and Marchantiopsida ^87, 103^—are circular molecules of 152 to 187 Kb in length that lack the *nad7* ^101, 102^ and *ccm* ^100^ genes in some species ( Figure 2). These mitogenomes have up to 20 to 24 unassigned ORFs. Green algae (Chlorophyta) mitogenomes vary between 16 and 130 Kb in length because of the presence of numerous tandem repeats ^88^. Whereas the mitogenomes of Prasinophytina are bigger and have the *nad10* gene as an addition to the angiosperm core set, the mitogenomes of Chlorophytina are smaller and show two different gene contents in Chlamydomonadales and Sphaeropleales, respectively ^88, 104^. The mitogenomes of Chlamydomonadales are generally linear with palindromic telomeres (but see 105) and lack many core genes ( *nad3*, *7*, *9*, *4L*; *cox2* and *3*; *atp1*, *4*, *6*, *8*, and *9*; all *rps* and *rpl*; and *tatC* ^88^), whereas those of Sphaeropleales have a more complete set of OXPHOS genes, lacking only *atp1*, *4*, and *8* ( Figure 2). All mitogenomes of green algae lack the *ccm*, *matR*, and *rrn5* genes ^88^. The number of *trn* genes varies between 23 and 27 in Prasinophytina but in Chlamydomonadales has been reduced to only three or four ^88^; it is remarkable that, in Sphaeropleales, all four *trnL* are missing ^104^. A notable feature of Chlorophytina mitogenomes is that *rrnL* and *rrnS* genes are fragmented. In Chlamydomonadales, these two genes are highly fragmented and the fragments are scattered across the chromosome, whereas in Sphaeropleales, the *rrnL* and *rrnS* consist of four and two fragments, respectively ^88, 104^. Another interesting feature of green algae mitogenomes is the evolutionary history of the *cox2* gene ^104^. This gene is complete in the mitogenomes of Prasinophytina and divided into two fragments, *cox2a* and *cox2b*, in Chlorophytina. Whereas in Sphaeropleales, the *cox2a* is in the mitogenome and the *cox2b* is in the nuclear genome, in Chlamydomonadales, both fragments have been transferred to the nuclear genome ^104^. Finally, it is important to note that Chlorophyta mitogenomes show important variations in the genetic code ^104^.

Green plants (Viridiplantae) and their unicellular close sister groups form the Archaeplastida. Within non-green plant Archaeplastida, the mitogenomes of Glaucophyta are circular and between 33 and 52 Kb in length ^106^. These mitogenomes have the *nad11* gene as an addition to the angiosperm core set but lack the *ccm*, *tatC*, and *matR* genes ( Figure 2). They also encode several *rpl* and *rps* genes as well as the *sdh3* and *4* genes ( Figure 2). There are two to 10 unassigned ORFs. Genes generally lack introns ^106^. These mitogenomes have *trn* genes sufficient to produce tRNAs that decode all codons but ACN (Threonine) ^106^. The mitogenomes of Rhodophyta are circular molecules of 25 to 30 Kb in length ^107, 108^. They lack some of the genes of the angiosperm core set ( *nad7* and *9*; *atp1*; *ccm*; and *matR*) and have few *rpl* and *rps* genes ( Figure 2).

## 4. Other eukaryote mitogenome structures

The characterization of mitogenomes from early diverging (mostly unicellular) lineages of eukaryotes (Amoebozoa, Heterokonta, Alveolata, Rhizaria, Cryptophyta, Centrohelida, Haptophyta, and Excavata) is key to understanding the evolutionary history of mitogenomes and their great diversity ^109^. The number of these mitogenomes that are being sequenced is growing fast but is still comparatively small given the high diversity of these lineages ( Figure 1).

Within Amoebozoa, the mitogenomes of Conosea ^110^ and Lobosea ^111^ have similar sizes of 29 to 58 Kb ( Figure 2). These circular mitogenomes generally have a core set of 19 OXPHOS subunit protein-coding genes ( *nad1–7*, *9*, *11*, *4L*; *cob*; *cox1–3*; *atp1*, *4*, *6*, *8*, and *9*) and several *rps* and *rpl* genes and unassigned ORFs ^110– 112^, although different species may have lost variously some of these genes; an extreme case is that of the free-living lobosean *Vannella croatica*, which lacks the *nad3*, *7*, and *9*; *atp1*, *4*, *6*, and *8*; and all *rps* and *rpl* genes ^113^. The *rrnS* and *rrnL* genes and 13 to 25 *trn* genes are present ^110– 112^. Most (if not all) genes are encoded in the same strand, and most *trn* genes are grouped into a single or few clusters. The *cox1* and *2* genes may be fused ^114^ and contain group I introns with *heg* genes ^113^. The *tufA* gene, which encodes for an elongation factor, has been reported in the lobosean *Vermamoeba vermiformis* (GenBank accession number: GU828005; unpublished). Interestingly, amoebozoan mitogenomes generally show little synteny and are highly divergent in sequence, even between individuals morphologically identified as belonging to the same species ^115^.

Within Straminopiles (or Heterokonta), the size and gene content of the mitogenomes of the main groups are generally conserved ( Figure 2). Several mitogenomes of Ochrophyta algae from families Chrysophyceae ^116^, Phaeophyceae ^117^, Eustigmatophyceae ^118^, and Bacillariophyceae (diatoms ^119^) have been sequenced. The circular genomes normally vary in size between 34 and 77 Kb, depending on intervening sequences of variable length. They code generally for the same protein-coding and *rrn* genes found in Amoebozoa mitogenomes except *atp1* (only present in Eustigmatophyceae ^118^). In addition, they encode the *rrn5*, *tatA* (not in all), and *tatC* genes. Remarkably, in Phaeopyceae, the *cox2* gene shows an in-frame insertion of about 3 Kb (1000 amino acids ^117^). The circular mitogenomes of Oomycota are compact and differ in size between the orders Peronosporales (38 Kb), Saprolegniales (47 to 49 Kb), and Pythiales (55 Kb ^120^). The gene content common to the three orders includes the same core set of OXPHOS system protein-coding genes found in Amoebozoa mitogenomes ^120^ ( Figure 2). An interesting addition to the RNA gene set is the presence of the *ssrA* gene, which encodes for a transfer-messenger RNA that releases translation complexes when stalled on mRNAs lacking a stop codon ^121^. The larger sizes of the Saprolegniales and Pythiales mitogenomes are due to duplication events in several genes ^120^. The mitochondrial genome of *Cafileria marina* represents the Bigyra ^122^. It is a circular molecule of 43 Kb, which has the same gene content as Amoebozoa mitogenomes ^122^. Remarkably, the *nad11* gene in many Heterokonta is split into two fragments; in several groups (for example, all Eustigmatophyceae, some Bacillariophyceae, and some Bygira), one of the fragments is relocated to the nuclear genome ^118^. The mitogenomes of *Blastocystis* are exceptional within Heterokonta as the species of this genus have mitochondrion-related organelles instead of true mitochondria ^123^. These mitogenomes are circular and of only 28 Kb in length, have a highly biased codon usage, and lack the *cox*, *cob*, and *atp* genes ^123^ ( Figure 2).

Within Alveolata, the mitogenomes of Ciliophora are linear (with long inverted repeats in the terminal regions) and have a relatively large size of 40 to 76 Kb ^124^ ( Figure 2). They are highly compact with small intergenic regions. They normally have a core set of 14 OXPHOS system protein-coding genes (they lack *nad11*, *cox3*, and *atp1*, *4*, *6*, and *8*) and a variable number of accessory *rps* and *rpl* genes ^124^ ( Figure 2). In addition, these mitogenomes have 21 or 22 unassigned ORFs, which encode proteins as large as 1300 amino acids ^125^. They have a reduced set of four to eight *trn* genes ^124^. Hence, nucleus-encoded tRNAs need to be imported to complete translation. Some of the genes, like *nad1* and the two *rrn* genes, are usually found fragmented ^125^. An exceptional case is that of *Nyctotherus ovalis* as the organelle genome is located in the hydrogenosome ^126^. This mitogenome of 48 Kb has the *nad* genes but lacks the *cox*, *cob*, and *atp* genes as well as most *rps*, *rpl*, and *trn* genes ^126^. The mitogenomes of apicomplexans are the smallest known (only 6 to 7 Kb ^127^), whereas those of dinoflagellates are relatively large (from 90 ^119^ to 326 Kb ^128^) ( Figure 2). The gene contents of the mitogenomes of apicomplexans and dinoflagellates are minimum with only three protein-coding genes ( *cox1* and *3* and *cob*), two fragmented rRNA genes and no *trn* genes ^128^ ( Figure 2). The rRNA fragments are coded on both strands of the mitogenome and not in linear order ^128^. In the *P. falciparum* mitogenome, up to 12 and 15 fragments need to be assembled (through a yet-unknown mechanism) into the ribosome to form the small (804 nt) and large (1233 nt) rRNAs, respectively ^129^. A certain degree of synteny of the fragmented genes is conserved among related taxa, and the fragmentation of the *rrn* genes was suggested to have occurred in the common ancestor of apicomplexans and dinoflagellates ^129^. In dinoflagellates, the diatom endosymbionts have mitogenomes with more canonical gene contents (see Ochrophyta mitogenomes ^119^).

Within Rhizaria, only mitogenomes of Cercozoa but not of Foraminifera and Radiolaria are available ( Figure 2). Within Cercozoa, the mitogenomes of *Lotharella oceanica* and *Bigelowiella natans* ^130^ and of *Spongospora subterranea* ^131^ and *Plasmodiophora brassicae* ^132^ represent Filosa and Endomyxa, respectively. The linear mitogenomes of *L. oceanica* and *B. natans* are 36 to 37 Kb in length and have terminal inverted repeats. They contain the same core set of OXPHOS system protein-coding genes of Amoebozoa except *nad11* and *atp4* as well as several *rps* and *rpl* genes. The *nad6* and *nad9* genes are duplicated in *L. oceanica*. No introns were found. Other genes involved in transcription, RNA processing, or protein import are missing ^130^. The circular mitogenome of *S. subterranea* is 38 Kb in length whereas that of *P. brassicae* is 115 Kb long, including a 12.5-Kb repeat ^132^. The gene contents of both mitogenomes are the same as those of Filosa plus *polB* and *rnpB* (Stjelja *et al*. ^132^ 2019). Up to 54 and five introns were found in *P. brassicae* and *S. subterranea*, respectively. Seven of the introns in *P. brassicae* were of group II. In total, 19 unassigned ORFs, most within introns, were inferred ^132^. The difference in number of introns, the long repeat, and intergenic regions may largely account for the different in lengths between the two mitogenomes.

The mitogenomes of Haptophyta ^133^, Centrohelida ^134^, and Cryptophyta ^135^ have been sequenced. (Sometimes these lineages have been grouped together within Hacrobia, although the monophyly of this group is highly controversial ^136^.) The mitogenomes of the Haptophyta are circular molecules of 29 to 34 Kb ( Figure 2). The intron-less genes are transcribed from the same strand. These mitogenomes have a reduced representation of OXPHOS system protein-coding genes, lacking *nad7*, *9*, and *11* and *atp1* and *8* and having only four and one *rps* and *rpl* genes, respectively ^133^. In contrast, the circular mitogenome of *Marophrys* sp. representing the Centrohelida is 113 Kb in length ^134^ and has a rather complete set of protein-coding genes, including *nad1*– *11*, *4L*; *cox1*– *3*; *cob*; *atp1*, *6*, *8*, and *9*; 12 *rps* and seven *rpl* genes; *sdh2*– *4*; *tufA*; *cox11*; and *polB* ( Figure 2). The genes *cox1* and *nad5* are partitioned whereas *nad9* is duplicated. There are 19 group I introns, most encoding *heg* genes. This mitogenome also has an *rpo* gene, which derives from integration of a mobile genetic element (linear plasmid) and encodes a single-subunit T7/T3-like RNA polymerase that is not involved in the transcription of the mitogenome. There are up to 12 unassigned ORFs. The mitogenomes of Cryptophyta are circular molecules ranging in size from 37 to 54 Kb ^135^ ( Figure 2). The mitogenomes contain the same set of OXPHOS system protein-coding genes reported in *Marophrys* sp.; a slightly reduced set of *rps* and *rpl* genes; and *sdh3* and *4* and *tatA* and *C* ^135^. The genes *cox1* and *cob* contain a group II intron in some species. Large syntenic blocks are conserved between species and a large repeat region is found ^135^.

Within Excavata, mitogenomes are extremely diverse ( Figure 2). The mitogenomes of Jakobida are the richest in gene content ^114, 137^ and the most similar to the transcriptional and translational machinery operons in bacterial genomes ^138^; those of Heterolobosea are compact and gene-rich ^139^; those of some Euglenozoa are arranged in specialized structures called kinetoplasts ^140^ and in Metamonada are simply absent as species either have highly reduced versions of mitochondria called mitochondrion-related organelles ^141, 142^ or completely lack them ^143^ because of their adaptation to anaerobic environments. Jakobid mitogenomes are mostly circular, of 67 to 100 Kb, and share a large core set of genes ^137, 144, 145^, which include the OXPHOS system protein-coding genes reported for *Marophrys* sp. plus *atp3* and *4* and the accessory genes encoding the RNA polymerase ( *rpoA–D*), several import transporters ( *secY* and *tatA* and *C*), proteins for binding of the heme *b* cofactor to cytochrome *c* through the maturation system I ( *ccmA–C* and *F*), and the RNAse P ( *rnpB*) ( Figure 2). The mitogenome of *Andalucia godoyi* has, in addition, the *cox15* encoding a protein involved in cytochrome *c* oxidase (COX) assembly ^137^. These mitogenomes also have the *ssrA* gene ^137^. The circular mitogenome of *Naegleria fowleri* as a representative of Heterolobosea is 49 Kb ^139^ but is almost as rich in genes as the jakobid mitogenomes ( Figure 2). Within Euglenozoa, the mitogenomes of *Euglena*, *Diplonema*, and *Tripanosoma* are radically different in organization but not in their reduced gene content ^140^. The short linear mitogenome molecules of 5 to 8 Kb in length of *Euglena* contain only the *nad1*, *4*, and *5*; *cob*; and *cox1–3* genes as well as fragmented and highly divergent *rrnL* and *rrnS* genes ^140^. The mitogenome of *Diplonema* is organized into about 80 to 100 circles of 6 to 7 Kb in length, each containing one or several (overlapping) gene fragments flanked by highly redundant non-coding regions ^140, 146^. The initial annotations of the mitogenome of *Diplonema* also reported the *nad7* and *8* and *atp6* genes in addition to the genes observed in *Euglena* ^140^. However, a recent study ^147^ demonstrated the presence of the *nad2*, *3*, *6*, *9*, and *4L* genes with highly divergent sequences. The mitochondrial protein-coding and *rrn* genes in *Diplonema* are highly fragmented, and gene pieces (or modules) are transcribed separately. The transcripts are end-processed to eliminate non-coding sequences and joined by an RNA ligase ^146^. Two types of RNA editing (one adding uridine tails at the 3′ ends of modules and the other producing C-to-U and A-to-I substitutions) restore ORFs post-transcriptionally ^146^. It is postulated that the origin of mitogenome and gene fragmentation in diplonemids could be related to a mobile element that proliferated and propagated ^146^. The molecular mechanism that ensures how chromosome loss is avoided during cell division is unknown ^146^.

The kinetoplasts of *Trypanosoma* are the most complex mitogenomes within Euglenozoa. The kinetoplast is a single disk-shaped structure composed of dozens of maxicircles of 20 Kb in length and thousands of minicircles of 1 Kb in length ^140^. The maxicircles have the genes found in *Diplonema* plus *rps12* and four unassigned ORFs. The minicircles encode small guide RNAs, which aid in post-transcriptional RNA editing ^140^. In Euglenozoa, all tRNAs must be imported from the cytosol.

## 5. Conclusions and perspectives

The last few years have witnessed an astounding increase in the number of sequenced mitogenomes. The advent of high-throughput sequencing techniques has extended mitogenome characterization to non-model, understudied eukaryotes and has facilitated the sequencing of large, fragmented, or repeat-rich mitogenomes otherwise intractable with traditional cloning, standard/long polymerase chain reaction (PCR), and sequencing methods. The rich database of mitogenomes available is still taxon-biased. The proportion of mitogenomes sequenced to the number of species per lineage is generally less than 10% (and in many instances less than 1%) and reaches only 21% in mammals, the best sampled case ( Figure 1). Yet a good-enough representation of most eukaryote groups is available not only to allow the consensus gene structures for the different groups to be inferred ( Figure 2) but also to recognize the astonishing diversity of exceptions to these consensuses. The early notion—based exclusively on the study of bilaterian species—that mitogenomes are one single chromosome and are circular and compact (of merely 16 Kb in length) has been largely superseded as the mitogenome structure of other eukaryotes has been unraveled ^13, 14, 47, 69, 125^. Mitogenomes are much more variable and dynamic than previously thought: their form can be circular, linear (with terminal inverted repeats), or branched; they can be organized into single or multipartite chromosomes; their sizes vary extensively from less than 10 Kb to more than 11 Mb and this is due mostly to changes in gene content and the presence of repeats in non-coding regions and of self-splicing introns. Their gene structure is also highly variable, including the presence of group I and II introns and the existence of partitioned genes (with the possibility of having one of the partitions in the nuclear genome).

The diversity of gene contents ( Figure 2) allows the main trends during the evolutionary history of mitochondria to be inferred ^109^. Alphaproteobacteria, the closest living group of mitochondria, have their genes encoding the subunits of the respiratory chain complexes and those encoding ribosomal proteins organized into operons, whose gene content and synteny are maintained in the mitogenomes of some unicellular eukaryotes, particularly jakobids and (to some extent) heteroloboseans. These bacterial operons also contain genes encoding proteins involved in protein transport, cytochrome *c* biogenesis, and DNA and RNA polymerization. All of these bacterial genes were inherited vertically into mitogenomes and later either maintained or eliminated variously depending of the evolutionary pathway, generating the great diversity in gene content, organization, and structure of mitogenomes which we observe in living eukaryotes ( Figure 2). The genes *cob*, *cox1* and *3*, and *rrnS* and *rrnL* are conserved throughout all known mitogenomes. The genes *nad1–6*, *4L*; *cox2*, *atp6*, *8*, and *9* are also highly conserved but are missing in some lineages, namely Apicomplexa, Dinoflagellata, and Euglenozoa. The genes *nad7* and *9*, *atp1* and *4*, *sdh3* and *4*, *tatC*, and *rrn5* are generally found in unicellular eukaryotes and Archeoplastida (with the exception of Rhodophyta) but not in fungi and animals. The genes *nad8* and *10*, *atp3*, and *sdh2* are restricted mostly to Excavata, Centrohelida, and Cryptophyta. The genes *rps* and *rpl* have been generally lost in animals, fungi (with the exception of *rps3*), Euglenozoa, and Alveolata. The presence of *rps16* and *rpl1*, *11*, *18–20*, *27*, *31–32*, and *34–35* is restricted to Jakobida and a few more lineages. The genes *ccmC*, *D*, and *F* are found in Jakobida, Heterolobosea, and Viridiplantae. In addition, the gene content of mitogenomes has been enriched repeatedly through scattered horizontal gene transfer of several genes of viral, bacterial, plastid, or nuclear origin. For instance, the presence of group I and II introns of bacterial/viral origin, which include genes that encode their own maturases, is widespread except in bilaterian animals, and the gene *matR* is prevalent in Viridiplantae.

The sequencing of mitogenomes has been boosted by high-throughput sequencing techniques. The genome skimming approach is particularly useful for sequencing mitogenomes ^148^. This technique consists of sequencing nuclear genomes at a low coverage. As a result, the recovery of high-copy fractions such as mitogenomes, which are present in thousands of copies per cell, is enhanced. Genome skimming looks particularly promising for obtaining mitogenomes from museum and herbarium-preserved old material ^149^. Several pipelines have been specifically designed to assemble mitogenomes from short reads ^150, 151^. Normally, the assembly is relatively straightforward, particularly if mitogenomes from closely related species are available and used as reference. However, the exceptions to this rule are plant mitogenomes, which are rather big and contain repeat regions prone to homologous recombination. These features have been a major drawback for long PCR amplification in the past and have seriously hindered assembly from short reads. In this regard, the use of technologies that produce long reads looks very promising for assembling plant mitogenomes ^152^ and in general for eliminating assembly chimaeras of short reads that may arise when using too-distant references. Mitogenome-encoded proteins have conserved domains that provide significant results in sequence similarity or hidden Markov model searches, which also facilitate (almost) automatic annotation. Hence, the perspective that the mitogenome database will grow exponentially in number and diversity in the coming years is well grounded and soon it will be possible targeting all key taxa representing main lineages of eukaryotes and, in particular, those groups of unicellular eukaryotes that are known only through metabarcoding approaches or those unknown that may live in extreme environments. The orthology of mitochondrial genes is relatively easy to assess and the sequences of these mitogenomes will allow species limits to be determined ^153^, cryptic species diversity to be detected ^115^, and robust trees to be reconstructed to help resolve ongoing phylogenetic controversies ^154^. The recovered trees could be used as evolutionary frameworks to compare changes in gene content and arrangement between sister taxa and infer the features of the mitogenomes of most recent common ancestors. This will provide us with an unprecedented view of the evolutionary transitions (gains, losses, and reorganizations) that occurred in mitogenomes during the diversification of eukaryotes and will help us understand the evolutionary mechanisms (for example, selection versus random drift) that triggered changes. As more nuclear genomes become sequenced, we will be able to understand the origin of mitochondrial intergenic regions and repeats on one side and the ultimate fate of the missing mitochondrial genes in the different mitogenomes. As more mitogenome organizations are characterized and combined with advance technologies such as single-cell transcriptomics, it will be possible to gain insights into the variety of replication, transcription, and translation (including RNA editing and changes in genetic code) processes occurring outside vertebrate mitogenomes and to further understand the evolution of the signaling occurring between the mitochondrial and the nuclear genomes.

## Appendix: Brief key to mitochondrial genes and corresponding protein functions

### 1. OXPHOS system proteins

The mitochondrial electron transport chain is composed of four (I–IV) complexes inserted in the inner mitochondrial membrane ^155^ that could be either physically connected in a supercomplex called the respirasome; connected only through redox reactions by two mobile molecules (the ubiquinone or Coenzyme Q and the cytochrome *c*) or dynamically shifting between the two extreme states ^156^. A fifth (V) complex is required to transform the proton pumping occurring in complexes I, II, and IV into ATP.

The NADH dehydrogenase (complex I) catalyzes the oxidation of NADH to NAD ^+^ and the transfer of electrons to ubiquinone. This complex of 44 protein subunits in humans is composed of a long hydrophobic membrane domain with the proton-pumping module and a hydrophilic peripheral domain with the NADH-binding and ubiquinone-binding modules ^157^. In bacteria, the complex I is encoded in the *nuo* operon, which consists of 14 genes ( *nuoA–N*). The genes *nuoA*, *H*, *J*, *K*, *L*, *M*, and *N* are homologs of mitochondrial genes ***nad3*, *1*, *6*, *4L*, *5*, *4*,** and ***2*,** respectively, which encode the chains inserted in the inner mitochondrial membrane. The genes *nuoB*, *C*, *D*, *G*, and *I* are homologs of mitochondrial genes ***nad10*, *9*, *7*, *11*,** and **8**, respectively, which encode subunits of the peripheral domain ^156^.

The succinate dehydrogenase (complex II) catalyzes the oxidation of succinate to fumarate with the reduction of ubiquinone to ubiquinol. One hydrophilic domain has two subunits: a flavoprotein (SdhA) that binds the succinate and an iron–sulfur protein (SdhB). There is also a hydrophobic domain anchored in the inner membrane with two subunits: the cytochrome *b*560 (SdhC) and another cytochrome *b* (SdhD). Genes ***sdh2*, *3*,** and ***4*** encode subunits B, C, and D, respectively.

The coenzyme Q-cytochrome *c* reductase (complex III) catalyzes the oxidation of ubiquinol and the transfer of electrons to cytochrome *c*. This complex is made of three proteins in bacteria and up to 11 in humans. The gene ***cob*** encodes for cytochrome *b*, an integral membrane protein with two heme groups which binds the ubiquinol ^155^.

The cytochrome *c* oxidase (COX) (complex IV) reduces molecular oxygen to water. The complex is fully integrated in the inner membrane and includes 13 to 17 protein subunits and several redox cofactors: a di-copper center (CuA) present in COX2, a heme group, and a binuclear heme *a*3-CuB center present in COX1 ^158^. The mitochondrial genes ***cox1*, *2*, and *3*** encode the corresponding subunits, which form the catalytic core and are the only ones present in bacteria.

The ATP synthase (complex V) produces ATP from ADP in the presence of a proton gradient across the membrane, which is generated by electron transport complexes of the respiratory chain. Complex V consists of two structural domains: F1, containing the extramembraneous catalytic core, and Fo, containing the membrane proton channel. Genes ***atp6*, *8*,** and ***9*** encode subunits of the Fo. Genes ***atp1*** and ***4*** encode the alpha and beta subunits of the F1, respectively.

### 2. Ribosomal components

Mitochondrial ribosomes are composed of small and large subunits ^159^. The genes ***rps1–4*, *7*, *8*, *10–14*,** and ***19*** and ***rrnS*** encode proteins and the rRNA (12S in animals, 15S in fungi, and 18S in plants), respectively, of the small subunit *.* The genes ***rpl2, 5, 6*, *11*, *14*, *16*, *19*, *31*** and ***rrnL*** encode proteins and the rRNA (16S in animals, 21S in fungi, and 26S in plants), respectively, of the large subunit ^159^. The gene ***rrn5*** encodes 5S rRNA of the large subunit in plants and most unicellular eukaryotes ^160^. In gammaproteobacteria, ribosomal protein genes are organized in operons combining *rps* and *rpl* genes as well as other genes such as *tufA*, *rpoA–D*, and *secY* ^138, 161^, a condition also found in some mitogenomes (for example, those of Jakobida).

### 3. Protein maturation

Cytochromes *c* are proteins with covalently attached heme *b* cofactors. The binding of the heme group relies on membrane-associated proteins named cytochrome *c* maturation systems, one of which is System I or Ccm ^162^. This system is organized into three functional modules: (1) transports the heme *b*, (2) has chaperoning function, and (3) performs the ligation. Genes ***ccmA–C*** encode three of the five subunits involved in module 1. Genes ***ccmFc*** and ***ccmFn*** encode proteins involved in module 3 ^162^.

Assembly of the redox cofactors requires the participation of a set of accessory proteins. In particular, gene ***cox11*** encodes a protein involved in copper insertion ^158^.

### 4. Transporters

Proteins can be secreted across a membrane either in their unfolded conformation (and later fold into their native structure) using the Secretory pathway or in their folded state using the Twin-arginine translocation (or SecY-independent) pathway. The genes involved in the two pathways are the *sec* and *tat* genes, respectively. The gene ***secY*** encodes for the central subunit of the secretory channel SecYEG. The genes ***tatA*** and ***tatC*** encode membrane-integrated subunits of the TAT channel ^163^.

### 5. Processing proteins

The family B of DNA polymerases, such as the DNA polymerase II of *Escherichia coli* or the T4 DNA polymerase, have replicative and 3′–5′ exonuclease proofreading activities ^164^. The ***polB*** (also referred to as *dpoB*) gene encodes these proteins.

The DNA-dependent RNA polymerase synthesizes RNA from a DNA template. In bacteria, the enzyme core is composed of five subunits: two α involved in assembly and transcriptional regulation, β and β′ involved in the catalysis, and ω involved in assembly, which are encoded by genes ***rpoA*, *B*,** and ***C*** ^165^ and ***Z*** ^166^, respectively. In addition, the σ transcription initiation factor, encoded by the ***rpoD*** gene, binds to the core, forming the holoenzyme. Archaeal and eukaryotic RNA polymerase core enzymes consist of 10 to 20 subunits ^167^.

The DNA mismatch repair system in gammaproteobacteria such as *E. coli* recognizes differences in the methylation state. This system has to recognize the mispair, propagate the signal, select the appropriate strand, excise it, and resynthesize a new one. Gene ***mutS*** encodes for an ABC-family ATPase that recognizes the mispair ^168^.

Ribonuclease P (RNase P) is an endonuclease that cleaves other RNA molecules at the junction between a single-stranded region and the 5′ end of a double-stranded region (for instance, in a tRNA precursor). The gene ***rnpB*** encodes for the RNA subunit ^169^.

The ***ssrA*** gene encodes a transfer-messenger RNA that participates in the so-called ribosome rescue pathway. This molecule releases translation complexes when stalled on mRNAs lacking a stop codon. First, it acts as a tRNA, binding to the stalled ribosomes, then as a mRNA, adding an ssrA peptide tag to the C-terminus of the nascent polypeptide chain, which is targeted for proteolysis ^170^.

Group I and II introns contain inside ***heg*** and ***matR*** genes, respectively, which encode RNA maturases. The maturases of group I have a homing endonuclease activity whereas those of group II have a reverse transcriptase activity. In addition, both have a ribozyme component, which catalyzes splicing ^54, 55^.

The gene ***tufA*** encodes for the protein synthesis elongation factor Tu (EF-Tu), which plays a central role in the elongation phase of protein synthesis by placing the aminoacyl-tRNA at the A site of the ribosome ^171^.

## Abbreviations

ATP, adenosine triphosphate; BP, base pair; CCM, cytochrome ***c*** maturation system; COB, apocytrochrome B; COX; cytochrome ***c*** oxidase; HEG, homing endonuclease; LSU, large ribosomal subunit; MAT, maturase; mRNA, messenger RNA; MUT, mutator; NAD, NAD dehydrogenase; nt, nucleotide; ORF, open reading frame; OXPHOS, oxidative phosphorylation; PCR, polymerase chain reaction; POLB, family B of DNA polymerases; RNP, ribonuclease P; RPL, ribosomal protein of the large subunit; RPO, DNA-dependent RNA polymerase; RPS, ribosomal protein of the small subunit; RRNA, ribosomal RNA; SAR, Stramenopiles, Alveolata, Rhizaria; SDH, succinate dehydrogenase; SEC, Secretory; SSR, 10Sa RNA; SSU, small ribosomal subunit; TAT, Twin-arginine translocation; tRNA, transfer RNA; TUF, TU elongation factor.
